# Pathogens and Their Effect on Exosome Biogenesis and Composition

**DOI:** 10.3390/biomedicines6030079

**Published:** 2018-07-23

**Authors:** Leandra B. Jones, Courtnee’ R. Bell, Kartz E. Bibb, Linlin Gu, Mamie T. Coats, Qiana L. Matthews

**Affiliations:** 1 Microbiology Program, Department of Biological Sciences, College of Science, Technology, Engineering and Mathematics, Alabama State University, Montgomery, AL 36104, USA; ljones@alasu.edu (L.B.J.); cbell@alasu.edu (C.R.B.); mcoats@alasu.edu (M.T.C.); 2 Department of Biological Sciences, College of Science, Technology, Engineering and Mathematics, Alabama State University, Montgomery, AL 36104, USA; kbibb@alasu.edu; 3 Division of Pulmonary, Allergy and Critical Care Medicine, Department of Medicine, University of Alabama at Birmingham, Birmingham, AL 35294, USA; linlingu_yz@hotmail.com; 4 Center for NanoBiotechnology Research and Department of Biological Sciences, Alabama State University, Montgomery, AL 36104, USA

**Keywords:** exosomes, *Trypanosoma cruzi*, bacteria, leishmaniasis, biomarker

## Abstract

Exosomes are nanosized membrane microvesicles (30–100 nm) that have the capability to communicate intercellularly and transport cell components (i.e., miRNA, mRNA, proteins and DNA). Exosomes are found in nearly every cell type (i.e., mast cells, dendritic, tumor, and macrophages). There have been many studies that have shown the importance of exosome function as well as their unique packaging and targeting abilities. These characteristics make exosomes ideal candidates to act as biomarkers and therapeutics for disease. We will discuss the biogenesis, composition, and relationship of exosomes with non-viral microbial infections including gram-negative bacteria, gram-positive bacteria, *Leishmania* and *Trypanosoma cruzi*.

## 1. Introduction

Extracellular vesicles (EVs) are secreted by cells and released into biological fluids in all living systems [1]. The EVs that are released can be pathogen or host-derived when an infection is present [2]. These vesicles are involved in complex intercellular communications [3] which are essential between cells. Cell-to-cell communication is necessary to maintain tissue and organ integrity, maintain homeostasis, and induce certain responses as a result of stimuli [4]. Many types of communication mechanisms between cells have been studied, including direct cell–cell connections (junctions), electrical stimuli, extracellular matrix interactions, and release of various chemical substances. Extracellular vesicles include exosomes, microvesicles, and apoptotic bodies [5] all of which carry a variety of molecules, including lipids, proteins and genetic material, such as DNA and non-coding RNA [6]. EVs vary in size and function and are released by different mechanisms from the cell. Originating from various subcellular compartments, the role of extracellular vesicles as regulators of biological information transfer is now a well-supported concept. Although, we now know the importance of EVs biologically and experimentally, we must now take painstaking efforts to isolate, purify, and characterize EV populations for diagnostic as well as therapeutic uses [7,8,9]. Careful consideration must be considered when evaluating EVs due to their overlapping size and composition.

## 2. Types of Extracellular Vesicles

### 2.1. Exosomes

Exosomes are the smallest type of EVs. These endosome-derived small membrane vesicles are approximately 30–100 nm in diameter [10,11]. Exosomes have the capacity to transfer proteins and nucleic acids through direct cell-to-cell contact as well as long-range signaling [10]. Most cells secrete exosomes being of endocytic origin [12]. Exosome interest has intensified due to their ability to be released from antigen-presenting cells to stimulate immune responses in vivo making them ideal for therapeutics [13]. They are also present in biofluids such as blood and the cerebrospinal fluid and play key roles in intercellular communication by transferring their materials between source and target cells under physiological and pathophysiological conditions [2]. An increasing body of studies have suggested that exosomes could potentially be used as biomarkers of infectious diseases with the possibility of preventing infection. A list of pathogens and their effect on hosts’ exosomes can be found in Table 1.

### 2.2. Microvesicles

Microvesicles (MVs), or membrane vesicles, or ectosomes, are substantially larger than exosomes ranging from 100–1000 nm. MVs differ from exosomes by the mechanisms of release and biogenesis. MVs are shed through outward budding and fission of membrane vesicles from the plasma membrane. In many ways, the fission resembles the abscission step in cytokinesis [23,24,25,26]. MV release also shares similarities with the mechanism of virus budding. Like exosomes, MVs carry a variety of molecules and are inducible, allowing their composition to be frequently enriched with molecules that are bioactive and whose production is specifically induced in response to a particular stimulus [23,24,25,26].

### 2.3. Apoptotic Bodies

Apoptotic Bodies (ABs) are the largest sized extracellular vesicle. ABs whose size varies between 1–5 µm are released by apoptotic cells as blebs [24,27,28]. AB release serves as a signal stimulating phagocytosis of apoptotic cells before induction of secondary necrosis [27,28]. ABs are enriched with various damage-associated molecular pattern proteins that can induce inflammation [27,28].

## 3. Exosome Biogenesis and Composition

Exosomes can be found in an array of eukaryotic fluids making them ideal candidates to serve as biomarkers. Exosomes have been found in the plasma [29], urine [29], cerebrospinal fluid [29], saliva [29], breast milk [30], amniotic [30], and bronchoalveolar lavage fluid [30]. Exosomes can be secreted by a large variety of cells, such as mast cells, dendritic cells, T cells, B cells, stem cells, astrocytes, endothelial cells, tumor cells, and epithelial cells [29,30,31]. Exosomes have several different surface molecules and are able to activate many cell receptors which allows them to participate in the exchange of materials between cells (i.e., proteins, lipids, carbohydrates, and pathogens) [23].

**Figure 1:**
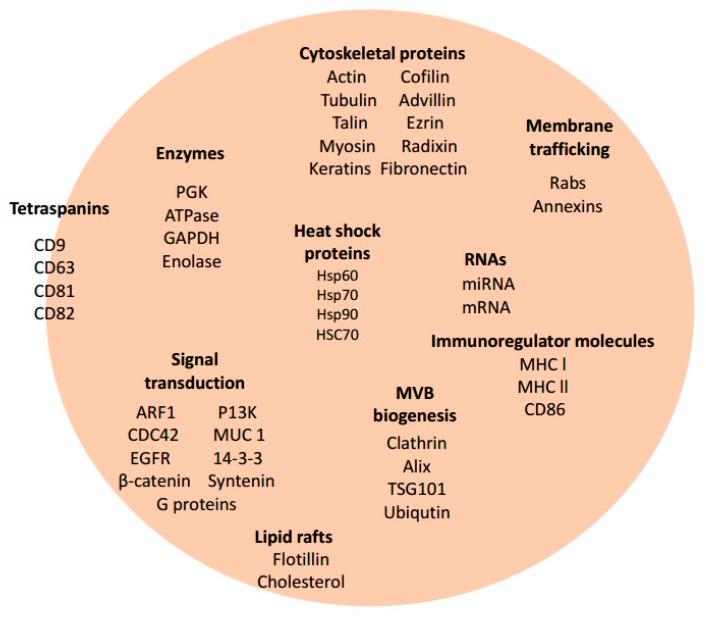
Composition of mammalian-derived exosomes. Proteomic, Biochemical, and Immunological investigations have identified many specific proteins and RNAs present in some exosomes. This is a limited representation of common molecules present within some mammalian-derived exosomes. Molecules illustrated here are grouped based on category function or protein class: Tetraspanins, Enzymes, Cytoskeletal proteins, Heat Shock proteins/chaperone proteins, Membrane trafficking proteins, Immunoregulator molecules, Lipid rafts, MVB biogenesis proteins, and RNAs. Some of these molecules are also found in bacteria derived-exosomes (Figure 2) and protozoan derived-exosomes (Figure 3).

The formation of intraluminal vesicles (ILVs) after the inward budding of the membranes of late endosomes begins exosome biogenesis [32]. Multivesicular bodies (MVBs) transit towards the cell surface, fuse with the plasma membrane, and release intraluminal vesicles outside of the cell and into the extracellular environment [33,34]. Exosomes are the only secreted cellular vesicles that are formed from internal membranes [33]. Exosomes are rich in lipid composition (cholesterol, sphingomyelin, and ceramide) and protein composition which deems them a distinctly different organelle from those of the cell [29]. Exosomes from varying cell types may have common proteins for membrane fusion and cytoskeleton regulation (Rab family, GTPase, Alix, endosomal sorting complex required for transport (ESCRT) (Figure 1). Exosome proteins can also be cell type-specific, only embedding proteins for a particular cell type [29].

The ESCRT is found on the membrane of exosomes and has been found to sort cargo into intraluminals, necessary for intraluminal formation and secretion [29,33,34]. ESCRT has five soluble multiprotein complexes (ESCRT-0, -I, -II, -III, and Vps4) [35]. The initiation of the mulitivesicular body pathway is caused by ESCRT-0. ESCRT-0 subsequently recruits ESCRT-I to the endosomal membrane, which in turn recruits the remaining members of the ESCRT machinery, ESCRT-II and -III [2]. It has been hypothesized that ESCRT-0, -II, and -III aid in the budding and release of the vesicle. Vps4 stops the sorting and vesicle formation process [3,32].

Exosomes contain various proteins which are displayed or expressed and are dependent on the cell origin in which the exosomes are derived. These proteins include chaperone proteins; heat shock proteins (HSPs) 60, 70, and 90; cytoskeletal proteins (actin, tubulin); fusion protein (flotillin); tetraspanins (CD9, CD37, CD53, CD63, CD81) [3,14,29,30]; and so on. (Figure 1). Tetraspanins are membrane proteins that contain large complexes found on the plasma membrane of exosomes making them model protein markers [33]. They are characteristically found within some exosomes and can be used to confirm the presence of exosomes. Tetraspanins facilitate cell fusion, migration, signaling, and cell-to-cell adhesion [36], and were originally identified in B lymphocytes; subsequent studies showed the presence of these proteins in additional cell types [3].

Tetraspanins (CD9, CD63, CD81 and CD82), co-stimulatory molecules (CD86), and adhesion molecules (CD11b, CD54, CD63) (Figure 1) are high in abundance within exosomes and mediate cargo sorting and ILV formation [37]. In addition, CD81 facilitates cargo sorting of tetraspanin ligands such as Rac GTPase [38]. CD9 tetraspanins are targeted for fusion with the plasma membrane [2]. CD55 and CD59 provide protection against the complement system which makes them a fundamental part of the stability of the exosome when circulating in body fluids [37]. Exosomes are rich in molecules that are involved in antigen presentation, such as CD1 and the major histocompatibility molecules (MHC) class I and II. MHC plays a significant role in immune-regulation by presenting antigenic peptides to T cells [37]. Other exosomal proteins include HSPs 70 and 90, which assist peptide loading onto MHC I and II and have a role in the cellular response to stressors. Acting as chaperone proteins, HSPs also assist in protein folding and trafficking [37]. Signal transduction proteins (G proteins and protein kinases) and proteolysis enzymes (which may increase cell migration) are also found in exosomes [37]. Exosomes do not enclose nuclear, endoplasmic, or mitochondrial proteins.

When exosomes are released from the plasma membrane containing cargo, they have the ability to transport those molecules (lipids, carbohydrates, miRNA, and proteins) throughout their extracellular environment [29]. The cargo that is carried is contingent on various factors such as cellular and environmental aspects [29]. The host cell is able to receive or uptake these molecules through juxtacrine, endocrine, and paracrine signaling [29]. The target cell is able to uptake this cargo from the exosomes by phagocytosis, direct fusion with the plasma membrane, or by receptor-mediated endocytosis [2]. Receptor-mediated endocytosis involves the direct binding of exosomes to receptors on the plasma membrane or the membrane of an endocytic organelle of the host cell [2]. Direct fusion allows for the cargo to be released directly into the cell while being fused to the receptors of plasma membrane [11].

## 4. Exosomes and Bacterial Infections

**Figure 2:**
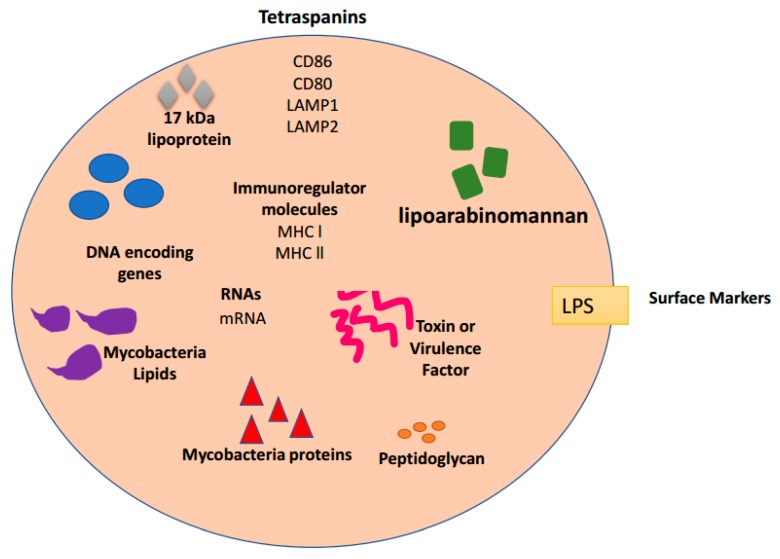
Composition of bacteria-derived exosomes. Proteomic, Biochemical, and Immunological investigations have identified many specific proteins and RNAs present in some exosomes. This is a limited representation of common molecules present within some bacteria-derived exosomes. Molecules illustrated here are grouped based on category function or protein class: Tetraspanins, Surface markers, Immunoregulator molecules, Mycobacteria proteins, Mycobacteria lipids, Toxins or virulence factors, DNA encoding genes, and RNAs. Some of these molecules are also found in protozoan derived-exosomes (Figure 3) and mammalian derived-exosomes (Figure 1).

Gram-negative bacteria have been shown to release vesicles for cell-to-cell communication with prokaryotic and eukaryotic cells [39]. Exosomes can play opposing roles in bacterial infections; these roles include activating immune response to prevent infection in an effort to protect the host [40] and/or assisting in spreading the infection [14]. In regards to preventing infection, the lipids, proteins, and carbohydrates found in exosomes can protect against subsequent infections by the insertion of microbial antigens into the host immune system [14,41]. In regards to assisting in the spread of the pathogen, lipids, proteins and carbohydrates found in exosomes can spread infection by activating the transcription of genes facilitating bacterial infection. Bacterial exosomes are able to transfer virulence factors (Figure 2), cause cytotoxicity, assist in bacterial invasion, and modulate the host immune response [41].

Giri and Schorey incubated exosomes from *M. Bovis* Bacillus Calmette-Guerin (BCG) infected cells and uninfected monocyte macrophages (J774) with splenocytes from infected mice [16]. Following incubation, exosomes from infected cells stimulated IFN-γ expression in CD8^+^ and CD4^+^ T cells whereas exosomes from noninfected cells did not induce IFN-γ [16]. CD69 (activation marker) expression was increased in T cells treated with exosomes from *M. Bovis* BCG infected J774 cells. Following those results, an in vivo study was completed to determine if exosomes had the ability to stimulate naïve T cells. The study design involved intranasally treated mice with exosomes ± adjuvant CpG, their findings showed a significant population of CD4^+^ and CD8^+^ T cells which produced IFN-γ [16]. This discovery proposed that exosomes have a substantial antigenic factor for both T cell populations [16]. Pro-inflammatory responses have been shown in exosomes from several types of bacterial infections including *Mycobacterium* species, *Klebsiella pneumoniae* and *Cryptococcus neoformans* (Table 1). These exosomes have also been shown to activate antigen-specific T cell response [42]. Khruh-Garcia and her associates set out to identify a biomarker panel for active Tuberculosis (TB) to be used as a diagnostic tool and to optimize a multiple reaction monitoring (MRM) method for marker detection [43]. They were able to identify 33 proteins targeted for detection in a prior study [43]. In their subsequent study, 20 of these 33 proteins targeted for detection were found in the exosomes of TB patients. Thus, tuberculosis infection can be diagnosed and classified (latent or active) by using exosomes [43]. Exosomes derived from bacteria-infected cells (Figure 2) could potentially be used for early identification of infectious pathogens that cannot be detected by standard methods such as PCR [44]. Previous studies have also shown that *M. avium* infected macrophages release exosomes containing a lipid-based cell wall component, that are then transferred to noninfected macrophages stimulating a pro-inflammatory response in latent macrophages (Table 1) [11]. By testing infected macrophages with *M. avium* infected macrophage exosomes, it was found that exosomes derived from bacterial components are released extracellularly and are able to conduct cell-to-cell communication via toll-like receptors [11]. Fleming et al. conducted a study on exosomal miRNA content, a function for bacterial pathogens [42]. Fleming and his colleagues found that infected THP-1 cell’s (*Yersinia pestis* or *Bacillus anthracis*) miRNAs were different in composition and packaging than uninfected cells.

*Pseudomonas aeruginosa* is responsible for only 400 deaths per year in healthcare settings. This bacterium can be treated with antibiotics; however, the bacterium has become resistant against some forms of antibiotic treatments [45]. The aforementioned pathogens secrete exosomes (Figure 2) that could potentially be used as a vaccine vehicle and/or used to learn more about the life cycle and pathogenesis of these pathogens [46]. Coakley and his colleagues demonstrated that targeting exosomes by vaccination with exosome-alum adjuvant provided immunity against helminths [47]. A similar strategy may be appropriate for Pseudomonas infections. Better knowledge of these exosomes may hold the answers to infection and disease progression. Exosomes ability to transfer information to uninfected cells for intercellular communication make them ideal candidates to battle diseases with no known treatment or vaccine.

## 5. Exosomes and Leishmaniasis Infections

Leishmanisis causes 20,000 to 30,000 deaths annually, an estimated 8 million people are affected by the disease worldwide [48]. Leishmaniasis is a parasitic disease found in the tropics, subtropics, and parts of Europe. Leishmaniasis is caused by a unicellular obligatory eukaryote [14]. There are three forms of Leishmanisis: visceral (affects the internal organs; sometimes fatal), cutaneous (skin ulcerations), and mucocutaneous (affects the mucous membranes of the nose, mouth, and throat cavities) [48]. Latent cases of Leishmaniasis may remain undiagnosed for years until the patient becomes immunocompromised. The patient will then develop fever, pancytopenia, and experience weight loss [48]. For the aforementioned reasons it is important to gain additional biological information concerning Leishmaniasis, this will aid in prevention and control of the disease.

**Figure 3:**
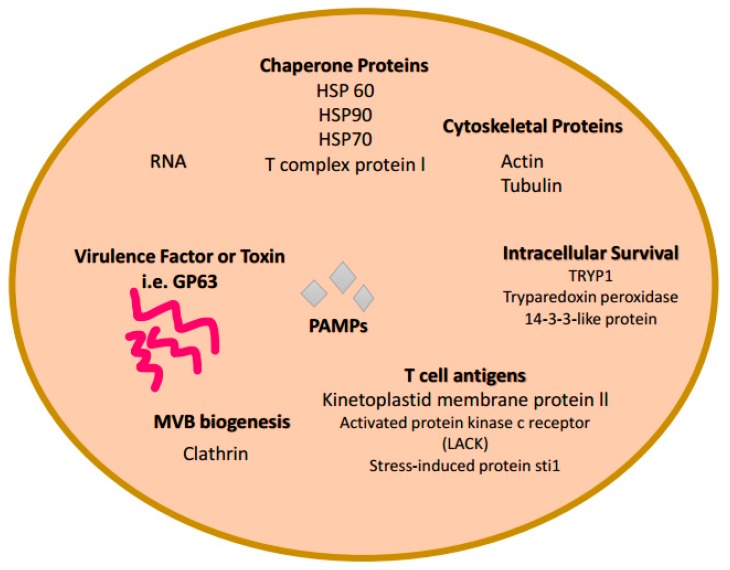
Composition of protozoa-derived exosomes. Proteomic, Biochemical, and Immunological investigations have identified many specific proteins and RNAs present in some exosomes. This is a limited representation of common molecules present within some protozoa-derived exosomes. Molecules illustrated here are grouped based on category function or protein class: Chaperone proteins, Cytoskeletal proteins, Toxins or virulence factors, MVB biogenesis proteins, and RNAs. Some of these molecules are also found in bacteria derived-exosomes (Figure 2) and mammalian derived-exosomes (Figure 1).

Previous studies have shown that membrane bound vesicles with exosome characteristics are released from *Leishmania* spp. and contain virulence factors (i.e., GP63, HSP 10, HSP 70, TRYP1, 14-3-3-like protein, stress induced protein sti1) [14,49] (Figure 3). The chaperone protein, HSP 100, which was identified in exosomes from *Leishmania*-infected hosts was capable of assisting with packaging of the parasite’s proteins into exosomes [2]. *Leishmania*’s major virulence factor, GP63, (Figure 3) is found in the exosomes of infected cells, which could possibly activate the immune responses in uninfected immune cells [50]. GP63 allows immune system related genes to express, which in turn signals MAP kinases and transcription factors (i.e., NK-K8) [2]. However, exosomes from *Leishmania*-infected macrophages have been found to suppress the immune system of its host due to the down regulation of pro-inflammatory genes and suppression of macrophage activation [51]. Silverman et al. were able to show that *Leishmania* exosomes selectively induce macrophage secretion of interlukin-8 in a dose–response study [49]. It has been found that *Leishmania*-derived exosomes are used as a vehicle for *Leishmania* protein secretion and translocation into macrophages [49].

Through exosome secretion *Leishmania* spp. interferes with both the innate and adaptive immune systems and attenuate them in the hosts [51]. *L. donovani* exosomes derived from HSP100k/o (lacks proteins found in the wild type) have been shown to stimulate the production of pro- and anti-inflammatory cytokines by monocytes and dendritic cells [14] (Table 1). These results were not shown in the wild type form of *Leishmania*. Linderstorm et al. have shown that by vaccinating the hosts with CAF01-pulsed HSP100k/o *L. donovani* exosomes, a lower count of the parasite was observed after infection [52]. This finding leads to the possibility of generating exosomes containing *Leishmania* antigens as a vaccine candidate in high risk areas.

## 6. Exosomes and *Trypanosoma cruzi* Infections

*Trypanosoma cruzi* is a protozoan parasite that causes Chagas disease in regions of the world near Central America, South America, and Mexico [53]. It has been estimated that 100 million people are at risk of infection because of this parasite [53]. Heart disease caused by Chagas is now considered an emerging global health problem. In the United States, undiagnosed Chagas disease is responsible for 30,000–45,000 cases of cardiomyopathy annually [54].

*T. cruzi* is mainly transmitted by blood feeding triatomine insects [55]. *T. cruzi* infects and multiplies in different organs [55]. Symptoms during the first 4–8 weeks are mild. *T. cruzi* amastigotes prefer cardiac and skeletal muscle tissue [55]. Many people are not aware of their infection and easily transmit the infection through blood donations [55].

Trypomastigotes form in the midgut of the Reduviid Bug and then transform into epimastigotes, the main replicating stage in the invertebrate host [56]. Epimastigotes migrate to the hindgut and differentiate into infective metacyclic trypomastigotes, which are excreted with the feces of the vector [57]. The insect bites the host, thereby transmitting metacyclic trypomastigotes [57,58]. They enter host cells through lysosome-mediated pathways then differentiate into trypomastigotes in the cells’ cytoplasm [56]. These trypomastigotes reproduce over 4–5 days and exit the cells into circulation where they can invade new cells and may be taken up again by the vector [57].

Exosomes deliver payloads that promote *T. cruzi* cell invasion and evasion from the host immune response [59]. MVs are shed at the plasma membrane while exosomes are formed within a network [60]. It has been reported that mammalian tissue culture trypomastigotes release MVs carrying virulence factors such as transialidase superfamily components [54,61,62,63,64,65,66,67] and that these extracellular vesicles are involved in Chagas disease progression by increasing heart parasitism and inflammation [68].

Infected mice and potentially infected cells, are targets for immune responses [67]. Where EVs were injected into BALB/c mice before infection, there was an increase in the number of *T. cruzi* trypomastigotes after infection [15,67]. Pronounced heart pathology also suggests EV involvement [69]. Monocytes were also induced by *T. cruzi* infection to produce high levels of EVs [69]. These EVs may protect against complement responses against *T. cruzi* [68]. The flagellar pocket and plasma membrane of *T. cruzi* released EVs during infection in host cells [53]. The same results were recorded in sandfly invasion with epimastigotes [67]. These studies further revealed that EVs released at the plasma membrane were MVs while those at the flagellar pocket may have been exosomes [53].

Several studies have attempted to identify surface and secreted components of *T. cruzi* implicated in host-cell invasion, which consists of a multi-step process involving various parasite and host-cell molecules. Nearly, 367 distinct proteins were identified as cargo of secreted vesicles and exosomes produced by *T. cruzi* [59]. Proteomic analyses classified those proteins into 16 categories, involving host-parasite interaction, signaling, transporters, carbohydrate metabolism, oxidation-reduction, and others [70] (Figure 3). The current evidence suggests that *T. cruzi* produces exosome-like particles from the free-living and intracellular stages, as well as triggers other cells for EV production, in order to modulate the host immune response. This is of utmost importance, in that, various forms of EVs (microvesicles and/or exosomes) are involved *T. cruzi* invasion and pathogenesis.

## 7. Conclusions

Exosomes are biological extracellular vesicles released from all cell types. These extracellular vesicles are involved in intercellular signaling transferring DNA, RNA, and proteins. They have been studied and found in 33 species of mammalian (Figure 1), viral, and bacterial cells (Figure 2). An exosome content database, Exocarta (exocarta.org), currently lists 9769 proteins, 3408 mRNAs, and 2838 microRNAs that have been found in various exosomes derived from several cell types and various organisms (Figure 3). Vesiclepedia (microvesciles.org) also cataloged protein, mRNAs, and lipid content of exosomes with the promise of continuously updating information as it is published by extracellular vesicle researchers.

Exosomes are released by cells experiencing normal physiological conditions, exogenous stress (drugs and alcohol) and during pathogenic challenge. Exosomal vesicles can be found in eukaryotic biological fluids making them ideal candidates acting as biomarkers. Exosome Diagnostics, Inc. (http://www.exosomedx.com/) has developed a revolutionary liquid biopsy platform that allows non-invasive detection of clinical biomarkers, one notable use being the recognition of prostate cancer from a simple urine sample [71].

An increasing body of studies have suggested that exosomes could potentially be used as biomarkers of infectious diseases with the possibility of preventing infection. Several pathogenic organisms are capable of releasing exosomes, some of which are listed in Table 1, fungus, *Cryptococcus neoforman* [72], *Leishmania major* [49], and *T. cruzi* [70]. A wealth of data suggests that *T. cruzi*-derived exosomes (Figure 3) play a role in the invasion of the host-cell and modulation of infection which antagonizes the host [59].

Exosomes have several attributes which allow them to be used as a vaccine vehicle. Exosome-mediated drug delivery might provide advantages that other systems do not provide, including limited or no undesired immunogenicity when self-derived exosomes are utilized [73], superior stability in the blood due to evasion of complement factors, efficient delivery of cargo into the cytosol of the target cell, and possibly less off-target effects due to the natural propensity of exosomes to target specifically [73]. The first evidence of exosome-mediated transfer of mRNAs and miRNAs was presented by Valadi et al., who showed that exosomes from mast cells contained large amounts of RNA [74]. This finding has tremendous implications due to the possibility of the role of exosomes in transporting genetic material leading to gene regulation and protein expression.

The notion that exosomes can be specifically targeted to cell types with low immunogenicity is an attractive avenue for pathogenic vaccinations. In this regard, Aline et al. performed ground-breaking research suggesting that host exosomes may be employed as a potential vaccine against toxoplasmosis (Table 1) [20]. A complete examination of exosomes derived from uninfected hosts and infected hosts is needed to elucidate mechanisms related to pathogenesis and new therapeutics.

## Figures and Tables

**Table 1:** Exosomes and their role in pathogenic infections.

Pathogen	Exosome Host	Role in Infection	References
*Leishmania donovani*	Macrophages Monoctyes (after IFN-γ treatment) Dendritic cells	• Suppression of immune system • Stimulation of IL-10, and suppression of IL-8 and TNF-α • Pro and anti-inflammatory response	[2,14]
*Trypanosoma cruzi*	Macrophages	• Evasion of host immune response • Division of exosomes • Apoptosis resistance	[15]
*Mycobacterium tuberculosis*	Macrophages, plasma	• Pro-inflammatory response	[16]
*Mycobacterium bovis*	Macrophages	• Pro-inflammatory response	[16]
*Mycobacterium avium*	Macrophages	• Pro-inflammatory response	[11]
*Bacillus anthracis*	Retinal pigment epithelial cells	• Transfer of lethal factor to uninfected cells	[17]
*Cryptococcus neoformans*	Macrophages	• Pro-inflammatory response	[18]
*Klebsiella pneumoniae*	Epithelial cells	• Pro-inflammatory response	[19]
*Toxoplasma gondii*	TAg-pulsed-DC2.4 Macrophages	• TH1 immune response • JNK Pathway activation	[20,21]
*Plasmodium yoelii*	Reticulocytes, Plasma	• Immune response modulation • Reticulocytosis • Change of cell tropism	[2,22]

## References

[1] Rilla K., Mustonen A.M., Arasu U.T., Harkonen K., Matilainen J., Nieminen P. (2017). Extracellular vesicles are integral and functional components of the extracellular matrix. Matrix Biol..

[2] Schorey J.S., Cheng Y., Singh P.P., Smith V.L. (2015). Exosomes and other extracellular vesicles in host-pathogen interactions. EMBO Rep..

[3] Raposo G., Stoorvogel W. (2013). Extracellular vesicles: Exosomes, microvesicles, and friends. J. Cell Biol..

[4] Lai C.P.-K., Breakefield X.O. (2012). Role of exosomes/microvesicles in the nervous system and use in emerging therapies. Front. Physiol..

[5] Yuan M.J., Maghsoudi T., Wang T. (2016). Exosomes mediate the intercellular communication after myocardial infarction. Int. J. Med. Sci..

[6] Andre F., Schartz N.E., Movassagh M., Flament C., Pautier P., Morice P., Pomel C., Lhomme C., Escudier B., Le Chevalier T. (2002). Malignant effusions and immunogenic tumour-derived exosomes. Lancet.

[7] Li P., Kaslan M., Lee S.H., Yao J., Gao Z. (2017). Progress in exosome isolation techniques. Theranostics.

[8] Zabeo D., Cvjetkovic A., Lasser C., Schorb M., Lotvall J., Hoog J.L. (2017). Exosomes purified from a single cell type have diverse morphology. J. Extracell. Vesicles.

[9] Momen-Heravi F., Balaj L., Alian S., Mantel P.Y., Halleck A.E., Trachtenberg A.J., Soria C.E., Oquin S., Bonebreak C.M., Saracoglu E. (2013). Current methods for the isolation of extracellular vesicles. Biol. Chem..

[10] Moldovan L., Batte K., Wang Y., Wisler J., Piper M. (2013). Analyzing the circulating micrornas in exosomes/extracellular vesicles from serum or plasma by qrt-pcr. Methods Mol. Biol..

[11] Bhatnagar S., Schorey J.S. (2007). Exosomes released from infected macrophages contain mycobacterium avium glycopeptidolipids and are proinflammatory. J. Biol. Chem..

[12] Honegger A., Schilling D., Sultmann H., Hoppe-Seyler K., Hoppe-Seyler F. (2018). Identification of e6/e7-dependent micrornas in hpv-positive cancer cells. Methods Mol. Biol..

[13] Shenoda B.B., Ajit S.K. (2016). Modulation of immune responses by exosomes derived from antigen-presenting cells. Clin. Med. Insights Pathol..

[14] Hosseini H.M., Fooladi A.A., Nourani M.R., Ghanezadeh F. (2013). The role of exosomes in infectious diseases. Inflamm. Allergy Drug Targets.

[15] Cestari I., Ansa-Addo E., Deolindo P., Inal J.M., Ramirez M.I. (2012). Trypanosoma cruzi immune evasion mediated by host cell-derived microvesicles. J. Immunol..

[16] Giri P.K., Schorey J.S. (2008). Exosomes derived from m. Bovis bcg infected macrophages activate antigen-specific cd4+ and cd8+ t cells in vitro and in vivo. PLoS ONE.

[17] Abrami L., Brandi L., Moayeri M., Brown M.J., Krantz B.A., Leppla S.H., van der Goot F.G. (2013). Hijacking multivesicular bodies enables long-term and exosome-mediated long-distance action of anthrax toxin. Cell Rep..

[18] Oliveira D.L., Nakayasu E.S., Joffe L.S., Guimaraes A.J., Sobreira T.J., Nosanchuk J.D., Cordero R.J., Frases S., Casadevall A., Almeida I.C. (2010). Characterization of yeast extracellular vesicles: Evidence for the participation of different pathways of cellular traffic in vesicle biogenesis. PLoS ONE.

[19] Lee J.C., Lee E.J., Lee J.H., Jun S.H., Choi C.W., Kim S.I., Kang S.S., Hyun S. (2012). Klebsiella pneumoniae secretes outer membrane vesicles that induce the innate immune response. FEMS Microbiol. Lett..

[20] Aline F., Bout D., Amigorena S., Roingeard P., Dimier-Poisson I. (2004). Toxoplasma gondii antigen-pulsed-dendritic cell-derived exosomes induce a protective immune response against t. Gondii infection. Infect. Immun..

[21] Li Y., Xiu F., Mou Z., Xue Z., Du H., Zhou C., Li Y., Shi Y., He S., Zhou H. (2018). Exosomes derived from toxoplasma gondii stimulate an inflammatory response through jnk signaling pathway. Nanomedicine.

[22] Martin-Jaular L., Nakayasu E.S., Ferrer M., Almeida I.C., Del Portillo H.A. (2011). Exosomes from plasmodium yoelii-infected reticulocytes protect mice from lethal infections. PLoS ONE.

[23] Lee Y., El Andaloussi S., Wood M.J. (2012). Exosomes and microvesicles: Extracellular vesicles for genetic information transfer and gene therapy. Hum. Mol. Genet..

[24] Rackov G., Garcia-Romero N., Esteban-Rubio S., Carrion-Navarro J., Belda-Iniesta C., Ayuso-Sacido A. (2018). Vesicle-mediated control of cell function: The role of extracellular matrix and microenvironment. Front. Physiol..

[25] Stahl P.D., Raposo G. (2018). Exosomes and extracellular vesicles: The path forward. Essays Biochem..

[26] D’Souza-Schorey C., Schorey J.S. (2018). Regulation and mechanisms of extracellular vesicle biogenesis and secretion. Essays Biochem..

[27] Chistiakov D.A., Orekhov A.N., Bobryshev Y.V. (2016). Cardiac extracellular vesicles in normal and infarcted heart. Int. J. Mol. Sci..

[28] Caruso S., Poon I.K.H. (2018). Apoptotic cell-derived extracellular vesicles: More than just debris. Front. Immunol..

[29] Corrado C., Raimondo S., Chiesi A., Ciccia F., De Leo G., Alessandro R. (2013). Exosomes as intercellular signaling organelles involved in health and disease: Basic science and clinical applications. Int. J. Mol. Sci..

[30] Zhou H., Cheruvanky A., Hu X., Matsumoto T., Hiramatsu N., Cho M.E., Berger A., Leelahavanichkul A., Doi K., Chawla L.S. (2008). Urinary exosomal transcription factors, a new class of biomarkers for renal disease. Kidney Int..

[31] Eldh M., Ekstrom K., Valadi H., Sjostrand M., Olsson B., Jernas M., Lotvall J. (2010). Exosomes communicate protective messages during oxidative stress; possible role of exosomal shuttle rna. PLoS ONE.

[32] Frydrychowicz M., Kolecka-Bednarczyk A., Madejczyk M., Yasar S., Dworacki G. (2015). Exosomes—Structure, biogenesis and biological role in non-small-cell lung cancer. Scand. J. Immunol..

[33] Meckes D.G., Raab-Traub N. (2011). Microvesicles and viral infection. J. Virol..

[34] Gyorgy B., Szabo T.G., Pasztoi M., Pal Z., Misjak P., Aradi B., Laszlo V., Pallinger E., Pap E., Kittel A. (2011). Membrane vesicles, current state-of-the-art: Emerging role of extracellular vesicles. Cell. Mol. Life Sci..

[35] Ha D., Yang N., Nadithe V. (2016). Exosomes as therapeutic drug carriers and delivery vehicles across biological membranes: Current perspectives and future challenges. Acta Pharm. Sin. B.

[36] Charrin S., Jouannet S., Boucheix C., Rubinstein E. (2014). Tetraspanins at a glance. J. Cell Sci..

[37] Beach A., Zhang H.G., Ratajczak M.Z., Kakar S.S. (2014). Exosomes: An overview of biogenesis, composition and role in ovarian cancer. J. Ovarian Res..

[38] Perez-Hernandez D., Gutiérrez-Vázquez C., Jorge I., López-Martín S., Ursa A., Sánchez-Madrid F., Vázquez J., Yáñez-Mó M. (2013). The intracellular interactome of tetraspanin-enriched microdomains reveals their function as sorting machineries toward exosomes. J. Biol. Chem..

[39] Hasegawa Y., Futamata H., Tashiro Y. (2015). Complexities of cell-to-cell communication through membrane vesicles: Implications for selective interaction of membrane vesicles with microbial cells. Front. Microbiol..

[40] Kruh-Garcia N.A., Wolfe L.M., Dobos K.M. (2015). Deciphering the role of exosomes in tuberculosis. Tuberculosis.

[41] De Toro J., Herschlik L., Waldner C., Mongini C. (2015). Emerging roles of exosomes in normal and pathological conditions: New insights for diagnosis and therapeutic applications. Front. Immunol..

[42] Fleming A., Sampey G., Chung M.C., Bailey C., van Hoek M.L., Kashanchi F., Hakami R.M. (2014). The carrying pigeons of the cell: Exosomes and their role in infectious diseases caused by human pathogens. Pathog. Dis..

[43] Kruh-Garcia N.A., Wolfe L.M., Chaisson L.H., Worodria W.O., Nahid P., Schorey J.S., Davis J.L., Dobos K.M. (2014). Detection of mycobacterium tuberculosis peptides in the exosomes of patients with active and latent m. Tuberculosis infection using mrm-ms. PLoS ONE.

[44] Maurin M. (2012). Real-time pcr as a diagnostic tool for bacterial diseases. Expert Rev. Mol. Diagn..

[45] Morita Y., Sobel M.L., Poole K. (2006). Antibiotic inducibility of the mexxy multidrug efflux system of pseudomonas aeruginosa: Involvement of the antibiotic-inducible pa5471 gene product. J. Bacteriol..

[46] Coakley G., Maizels R.M., Buck A.H. (2015). Exosomes and other extracellular vesicles: The new communicators in parasite infections. Trends Parasitol..

[47] Coakley G., McCaskill J.L., Borger J.G., Simbari F., Robertson E., Millar M., Harcus Y., McSorley H.J., Maizels R.M., Buck A.H. (2017). Extracellular vesicles from a helminth parasite suppress macrophage activation and constitute an effective vaccine for protective immunity. Cell Rep..

[48] World Health Organization Leishmaniasis. http://www.who.int/leishmaniasis/en/.

[49] Silverman J.M., Clos J., de’Oliveira C.C., Shirvani O., Fang Y., Wang C., Foster L.J., Reiner N.E. (2010). An exosome-based secretion pathway is responsible for protein export from leishmania and communication with macrophages. J. Cell Sci..

[50] Chaudhuri G., Chaudhuri M., Pan A., Chang K.P. (1989). Surface acid proteinase (gp63) of leishmania mexicana. A metalloenzyme capable of protecting liposome-encapsulated proteins from phagolysosomal degradation by macrophages. J. Biol. Chem..

[51] Silverman J.M., Clos J., Horakova E., Wang A.Y., Wiesgigl M., Kelly I., Lynn M.A., McMaster W.R., Foster L.J., Levings M.K. (2010). Leishmania exosomes modulate innate and adaptive immune responses through effects on monocytes and dendritic cells. J. Immunol..

[52] Schnitzer J.K., Berzel S., Fajardo-Moser M., Remer K.A., Moll H. (2010). Fragments of antigen-loaded dendritic cells (DC) and DC-derived exosomes induce protective immunity against leishmania major. Vaccine.

[53] Udoko A.N., Johnson C.A., Dykan A., Rachakonda G., Villalta F., Mandape S.N., Lima M.F., Pratap S., Nde P.N. (2016). Early regulation of profibrotic genes in primary human cardiac myocytes by trypanosoma cruzi. PLoS Negl. Trop. Dis..

[54] Leslie M. (2011). Infectious diseases. Drug developers finally take aim at a neglected disease. Science.

[55] Villalta F., Dobish M.C., Nde P.N., Kleshchenko Y.Y., Hargrove T.Y., Johnson C.A., Waterman M.R., Johnston J.N., Lepesheva G.I. (2013). Vni cures acute and chronic experimental chagas disease. J. Infect. Dis..

[56] Bern C., Kjos S., Yabsley M.J., Montgomery S.P. (2011). Trypanosoma cruzi and chagas’ disease in the united states. Clin. Microbiol. Rev..

[57] Gascon J., Bern C., Pinazo M.J. (2010). Chagas disease in spain, the united states and other non-endemic countries. Acta Trop..

[58] Matthews Q.L., Farrow A.L., Rachakonda G., Gu L., Nde P., Krendelchtchikov A., Pratap S., Sakhare S.S., Sabbaj S., Lima M.F. (2016). Epitope capsid-incorporation: New effective approach for vaccine development for chagas disease. Pathog. Immun..

[59] Borges B.C., Uehara I.A., Dias L.O., Brígido P.C., da Silva C.V., Silva M.J. (2016). Mechanisms of infectivity and evasion derived from microvesicles cargo produced by trypanosoma cruzi. Front. Cell. Infect. Microbiol..

[60] Van der Heyde H.C., Gramaglia I., Combes V., George T.C., Grau G.E. (2011). Flow cytometric analysis of microparticles. Methods Mol. Biol..

[61] Affranchino J.L., Ibanez C.F., Luquetti A.O., Rassi A., Reyes M.B., Macina R.A., Aslund L., Pettersson U., Frasch A.C. (1989). Identification of a trypanosoma cruzi antigen that is shed during the acute phase of chagas’ disease. Mol. Biochem. Parasitol..

[62] Goncalves M.F., Umezawa E.S., Katzin A.M., de Souza W., Alves M.J., Zingales B., Colli W. (1991). Trypanosoma cruzi: Shedding of surface antigens as membrane vesicles. Exp. Parasitol..

[63] Umezawa E.S., Shikanai-Yasuda M.A., Stolf A.M. (1996). Changes in isotype composition and antigen recognition of anti-trypanosoma cruzi antibodies from acute to chronic chagas disease. J. Clin. Lab. Anal..

[64] Jazin E.E., Bontempi E.J., Sanchez D.O., Aslund L., Henriksson J., Frasch A.C., Pettersson U. (1995). Trypanosoma cruzi exoantigen is a member of a 160 kda gene family. Parasitology.

[65] Mack M., Kleinschmidt A., Bruhl H., Klier C., Nelson P.J., Cihak J., Plachy J., Stangassinger M., Erfle V., Schlondorff D. (2000). Transfer of the chemokine receptor ccr5 between cells by membrane-derived microparticles: A mechanism for cellular human immunodeficiency virus 1 infection. Nat. Med..

[66] Mantel P.Y., Marti M. (2014). The role of extracellular vesicles in plasmodium and other protozoan parasites. Cell. Microbiol..

[67] Pinho R.T., Vannier-Santos M.A., Alves C.R., Marino A.P., Castello Branco L.R., Lannes-Vieira J. (2002). Effect of trypanosoma cruzi released antigens binding to non-infected cells on anti-parasite antibody recognition and expression of extracellular matrix components. Acta Trop..

[68] Trocoli Torrecilhas A.C., Tonelli R.R., Pavanelli W.R., da Silva J.S., Schumacher R.I., de Souza W., Silva N.C., de Almeida Abrahamsohn I., Colli W., Manso Alves M.J. (2009). Trypanosoma cruzi: Parasite shed vesicles increase heart parasitism and generate an intense inflammatory response. Microbes Infect..

[69] Cestari I., Ramirez M.I. (2010). Inefficient complement system clearance of trypanosoma cruzi metacyclic trypomastigotes enables resistant strains to invade eukaryotic cells. PLoS ONE.

[70] Bayer-Santos E., Aguilar-Bonavides C., Rodrigues S.P., Cordero E.M., Marques A.F., Varela-Ramirez A., Choi H., Yoshida N., da Silveira J.F., Almeida I.C. (2013). Proteomic analysis of trypanosoma cruzi secretome: Characterization of two populations of extracellular vesicles and soluble proteins. J. Proteome Res..

[71] Overbye A., Skotland T., Koehler C.J., Thiede B., Seierstad T., Berge V., Sandvig K., Llorente A. (2015). Identification of prostate cancer biomarkers in urinary exosomes. Oncotarget.

[72] Rodrigues M.L., Nakayasu E.S., Oliveira D.L., Nimrichter L., Nosanchuk J.D., Almeida I.C., Casadevall A. (2008). Extracellular vesicles produced by cryptococcus neoformans contain protein components associated with virulence. Eukaryot. Cell.

[73] Kooijmans S.A., Vader P., van Dommelen S.M., van Solinge W.W., Schiffelers R.M. (2012). Exosome mimetics: A novel class of drug delivery systems. Int. J. Nanomed..

[74] Valadi H., Ekstrom K., Bossios A., Sjostrand M., Lee J.J., Lotvall J.O. (2007). Exosome-mediated transfer of mrnas and micrornas is a novel mechanism of genetic exchange between cells. Nat. Cell Biol..

